# Clinical Informed Consent and ABA

**DOI:** 10.1007/s40617-023-00902-0

**Published:** 2024-01-08

**Authors:** Abraham Graber, Allison Maguire

**Affiliations:** 1 Division of Bioethics, Nisonger Center Affiliate Faculty, Department of Biomedical Education and Anatomy, Wexner College of Medicine, The Ohio State University, Columbus, OH USA; 2 Department of Athletics, University of Texas at San Antonio, San Antonio, TX USA

**Keywords:** Informed consent, Ethics, Law

## Abstract

The practice of clinical informed consent in America is governed by over 100 years of case law. Although predominant ethics resources for behavior analysts offer some guidance regarding the provision of clinical informed consent, such guidance remains limited. The goal of this article is thus to expand the contemporary literature on clinical informed consent in behavior analysis by providing a historical and contemporary guide to relevant case law. The article will highlight seminal moments in the history of case law regarding clinical informed consent, discuss their applicability to the process of clinical informed consent in behavior analysis, and provide an enhanced understanding of the ethical and legal obligations related to informed consent in the therapeutic context.

**Supplementary Information:**

The online version contains supplementary material available at 10.1007/s40617-023-00902-0.

## Introduction

The Nuremberg Code and the Belmont Report jointly serve as the foundations for the contemporary Western approach to both clinical and research ethics (Cassell, 2000; Shuster, 1997). These documents, respectively a reaction to the horrors of Nazi experimentation and the abuses of the Tuskegee Syphilis Study, mark a sea change in the received understanding of biomedical ethics (Cassell, 2000; Shuster, 1997). *Parentalism*,^1^ the view that the health-care provider knows what is in the patient’s best interest and should thus make decisions on behalf of the patient, was previously dominant (Emanuel & Emanuel, 1992; Katz, 1984). The abuses of the Tuskegee Syphilis Experiment and the physician perpetrated atrocities of the Holocaust offered indisputable evidence that health-care providers could not be universally entrusted to make decisions on patients’ behalf.

In the wake of the Nuremberg Code and the Belmont Report, parentalism gave way to the emphasis on respect for patient autonomy that characterizes contemporary Western health-care (Katz, 1984). Two distinct justifications are given for this new focus on patient autonomy. First, decisionally capacitated adults are taken to have a fundamental right to make determinations about what is done to their bodies (Beauchamp & Childress, 2019). Second, patients are uniquely positioned to both know what is in their best interest and to safeguard their own well-being (Eyal, 2019). Whereas the first of these justifications views autonomy as good-for-its-own-sake, the latter sees respect for autonomy as a means of protecting patient welfare.

The theoretical shift toward respect for patient autonomy was reflected in the therapeutic context by a renewed focus on *informed consent*. Informed consent must meet three conditions. First, the patient must be *informed*. That is, the patient must have access to all requisite information about available interventions and outcomes (Appelbaum, 2007; Beauchamp & Childress, 2019; Eyal, 2019). Second, the patient must be *decisionally capacitated* and/or *decisionally competent*.^2^ In other words, the patient must have the mental and emotional acuity necessary for decision-making (Appelbaum, 2007; Beauchamp & Childress, 2019; Eyal, 2019). Finally, the patient must be free from coercion and undue inducement (Appelbaum, 2007; Beauchamp & Childress, 2019; Eyal, 2019). Put another way, the patient must not be making their decision in response to a (real or perceived) threat nor in response to the (real or perceived) promise of benefits that they would otherwise not be able to access, other than the expected outcomes of the intervention.

Informed consent is generally required before a clinical intervention can be legally or ethically provided (Beauchamp & Childress, 2019). This remains the case even in those instances where a client lacks decisional capacity/competence. In such cases, a *proxy decision-maker* must make decisions on behalf of the client (Appelbaum, 2007; Beauchamp & Childress, 2019). A client’s proxy decision-maker will generally be a parent, legal guardian, or family member; however, in some cases a health-care provider may serve in this role (Beauchamp & Childress, 2019). When a client lacks decisional capacity/competence, the health-care provider’s obligation to engage in the clinical informed consent process with the client becomes an obligation to engage in the clinical informed consent process with the proxy decision-maker.

Recent behavior analytic literature has highlighted the importance of assent in therapeutic contexts (Flowers & Dawes, 2023; Breaux & Smith, 2023). Assent and informed consent are closely related. The key difference is that whereas a client or proxy decision-maker must be decisionally capacitated/competent in order to provide informed consent, assent occurs when a client who lacks decisional capacity/competence agrees to an intervention or assessment (Beauchamp & Childress, 2019). Informed consent is both legally and ethically necessary prior to implementing an intervention or assessment (Beauchamp & Childress, 2019). By contrast, there is no legal requirement to receive assent before implementing a therapy. In addition, although some authors argue that assent is an ethical necessity in therapeutic contexts (Flowers & Dawes, 2023; Breaux & Smith, 2023), this perspective is contested by other authors (Wasserman et al., 2019). Because this article focuses on the legal requirements that govern the provision of informed consent, and because there is no legal requirement to receive assent in therapeutic contexts, this article will not engage with questions about the appropriate role of assent in behavior analytic practice.

The requirement that a decision-maker provide informed consent serves as the primary protection against parentalism in therapeutic contexts (Beauchamp & Childress, 2019; Eyal, 2019). Necessitating informed consent empowers the client with ultimate decisional authority regarding what can be done to their body; the health-care provider cannot permissibly (legally or ethically) force their will onto the patient. Furthermore, requiring *informed consent*—rather than mere consent—guarantees that clients have the information necessary to make health-care decisions that genuinely reflect their values and preferences (*Salgo* v. *Leland Stanford Jr. Univ. Bd. of Trs*, 1957).

Informed consent is best understood not as an act, for example, signing a document that gives permission for intervention, but rather as a process (Brody, 1989). Informed consent as a process captures two key features of how informed consent should proceed. First, informed consent should be dynamic rather than static. It is not adequate for a health-care provider to merely recite relevant information and leave the rest to the decision-maker. Rather, the health-care provider should provide information and, in addition, invite and respond to queries, identify, and rectify potential misunderstandings, and otherwise support the decision-maker in making their choice (Behavior Analyst Certification Board [BACB], 2020; Brody, 1989; Emanuel & Emanuel, 1992). Informed consent as a process, rather than an act, further captures the on-going nature of informed consent. Where continuing care is provided, as in early intensive behavioral intervention (EIBI), a decision-maker must provide informed consent at each decision point (Brody, 1989).

Far from being a mere formality that must be observed in order to avoid litigation, informed consent is taken to be a core feature of the practice of good clinical practice:

Informed consent, properly understood, must be considered an essential ingredient of good patient care, and a physician who lacks the skills to inform patients appropriately and obtain proper consent should be viewed as lacking essential medical skills necessary for practice. (Brody 1989, p. 5)

A number of behavior analytic sources provide valuable guidance regarding the practice of informed consent in the clinical context. For example, the *Ethics Code for Behavior Analysts* (BACB, 2020) enumerates a range of information that must be disclosed as part of the clinical process of informed consent:

1) the purpose of the services . . . ; 2) the expected time commitment and procedures involved; 3) the right to decline to participate or withdraw at any time without adverse consequences; 4) potential benefits, risks, discomfort, or adverse effects; 5) any limits to confidentiality or privacy; . . . 7) whom to contact for questions or concerns at any time; and 8) the opportunity to ask questions and receive answers. (BACB, 2020, p. 7)

The *Ethics Code for Behavior Analysts* further stipulates that, “Behavior analysts are responsible for knowing about and complying with all conditions under which they are required to obtain informed consent from clients. . .” (BACB, 2020, p. 11).

Other available behavior analytic resources have similar strengths. For example, in *Ethics for Behavior Analysts* (4^th^ ed.), using the definition of “informed consent” provided by the *Ethics Code for Behavior Analysts*, Bailey and Burch (2022) discuss the value and implementation of informed consent across a range of research and clinical contexts.

In a similar vein, Brodhead et al.’s (2018) *Practical Ethics for Effective Treatment of Autism Spectrum Disorder* features a carefully considered case study of the informed consent process involving an English-speaking behavior analyst and a Spanish-speaking decision-maker. This case study has, however, been dropped from the second edition of the book, which now lacks an in-depth exploration of the clinical practice of informed consent (Brodhead et al., 2022).

Susha and Najdowski’s (2021) *A Workbook of Ethical Case Scenarios in Applied Behavior Analysis* includes case scenarios related to the provision of informed consent; however, all of the relevant case scenarios focus on informed consent in a research context. The book of scenarios does not include any cases focused on informed consent in the clinical context nor is there any discussion—outside of the *Ethics Code for Behavior Analysts*, which is included in the book—of clinical informed consent.

Finally, Beirne and Sadavoy’s (2022) edited volume, *Understanding Ethics in Applied Behavior Analysis: Practical applications* (2nd ed.), offers a robust discussion of informed consent in a number of different places and further distinguishes between informed consent in clinical and research contexts. Nonetheless, their discussion of informed consent is tightly tied to the presentation of informed consent in the *Ethics Code for Behavior Analysts* (BACB, 2020) and does not indicate that there may be disclosure requirements exceeding those indicated by that document (Beirne & Sadavoy, 2022).

Though the primary authoritative texts in the field of applied behavior analysis (ABA) offer behavior analysts valuable guidance regarding the practice of clinical informed consent, there is currently no resource designed to familiarize behavior analysts with the broader case law governing the practice of clinical informed consent in the United States. Such a guide is of particular importance because case law regarding informed consent includes explicit requirements that are not enumerated in extant behavior analytic texts. It is important that behavior analysts be familiar with these requirements both because they are legally binding and because, as the *Ethics Code for Behavior Analysts* highlights, behavior analysts are professionally “responsible for knowing about and complying with all conditions under which they are required to obtain informed consent from clients. . .” (BACB, 2020, p. 11).

This article aims to extend the contemporary discussion of clinical informed consent in behavior analysis by providing a guide to the history and contemporary status of the case law governing the provision of clinical informed consent. In what follows we will highlight key moments in the case law of clinical informed consent and, in many cases, supplement this historical narrative with brief discussions that illustrate the relevance of legal precedent to the clinical practice of ABA. The ultimate goal is to provide behavior analysts with a guide that will afford a more complete understanding of the legal requirements on clinical informed consent.

It is important to note that this article focuses on informed consent in the clinical or therapeutic context. We will not discuss the legal requirements on informed consent in the research context. Though related, the case law regarding clinical informed consent is distinct from the case law regarding informed consent in the research context (Grimm, 2007). Furthermore, behavior analysts engaging in human subjects research who are unsure about the legal requirements on informed consent in the research context should have ready access to guidance in the form of their institutional review board (IRB). The need for guidance regarding the case law governing informed consent is thus unique.

Given the breadth and depth of the legal precedents regarding informed consent, we cannot provide an exhaustive review of the case law governing informed consent. Furthermore, the laws governing informed consent in any particular geographical area in the United States are determined by a mix of state law, federal law, case law arising from court decisions, and statutory law written by legislatures. Legal requirements on informed consent will thus vary depending on the state and federal district in which one practices. As a result, there is no one-size-fits-all guide to the legal requirements of clinical informed consent. Depending on the stringency of the legal requirements governing informed consent in the locality where the reader practices, the following discussion can be understood either as highlighting (some of the) minimum legal requirements on the provision of informed consent *or* as merely best practice guidelines that may go beyond what is strictly required by law.

## Why Review Legal Cases?

The value of the approach to informed consent found in this article may not be immediately apparent. Why consider the history of case law regarding clinical informed consent, rather than merely providing a checklist behavior analysts can use to guide their practice? Our approach is, in part, informed by resistance to the idea that informed consent is reducible to completing a checklist and receiving a signature. There are, however, additional practical reasons that support our approach.

First, as will become clearer in the following discussion, the legal standards that govern the practice of informed consent vary widely depending on the state and federal district in which one practices. As such, it is impossible to provide a single checklist that accurately captures the legal requirements on informed consent for all behavior analysts (this is true even if one limits the discussion to behavior analysts practicing in the United States). Thus, any guidelines designed to help behavior analysts follow the legal requirements on informed consent risks being misleading unless presented in the relevant historical context.

Second, and more important, the legal requirements on informed consent have continued to expand, including substantial changes to the person required to provide informed consent as recently as 2017 (Lynch et al., 2018).^3^ In each instance where a court further expands the legal requirements involved in the process of informed consent, a health-care provider is found liable for failing to obtain informed consent *despite* fulfilling all of the legal requirements on informed consent recognized by the courts at the time the relevant intervention was implemented. As such, any guidelines related to the legal requirements on informed consent can provide only limited assurances to the behavior analyst.

Court decisions that expand the legal requirements on informed consent are not, however, arbitrary. Rather, they reflect the judgment of the courts about the applicability of preexisting principles to novel contexts. Especially given the lack of legal precedent regarding the process of informed consent in behavior analytic contexts, it is thus important for behavior analysts to be familiar with the legal principles underlying the expanding legal requirements on informed consent, as such familiarity can afford behavior analysts guidance unavailable through a static checklist. A review of the legal cases that have gradually expanded the legal requirements on informed consent provide multiple exemplars of the application of the relevant legal principles and thus serves to help behavior analysts develop the necessary repertoire of verbal behaviors.

In the discussion section of the article (and in Appendix 1), we will provide a resource aimed at helping behavior analysts fulfill the legal requirements on informed consent. The impatient reader may skip ahead to that resource. However, for the above reasons, we recommend against doing so. Merely following a checklist can never replace mastery of the “essential . . . skills necessary for practice” (Brody, 1989, p. 5); the practice of informed consent is no exception to this rule.

## The Genesis of Informed Consent Case Law and Concomitant Ambiguity for ABA

Informed consent case law in America originated with the 1914 case *Schloendorff* v. *Society of New York Hospital***.** In 1908, Mary Schloendorff suffered from stomach pain and became an inpatient at the Society of New York Hospital. Dr. Barlett, her treating physician, discovered a lump potentially responsible for Ms. Schloendorff’s symptoms. Ms. Schloendorff was advised that, in order for the nature of the lump to be determined, she would need to undergo a surgical operation under general anesthetic. Ms. Schloendorff testified that she consented to the operation but also that she explicitly refused consent for anything beyond a diagnostic procedure. While Ms. Schloendorff was under general anesthetic, the tumor was removed. Ms. Schloendorff subsequently developed gangrene which required the amputation of several of her fingers. Ms. Schloendorff sued on the grounds that she had not provided consent for the removal of the tumor (*Schloendorff* v. *Society of New York Hospital*, 1914).

The Court ruled in Ms. Schloendorff’s favor, writing “Every human being of adult years and sound mind has a right to determine what shall be done with his own body; and a surgeon who performs an operation without his [sic] patient's consent, commits an assault, for which he [sic] is liable in damages” (§129–130). In its ruling the Court set the precedent that carrying out a medical intervention without appropriate consent constitutes a violation of laws forbidding battery.

The precedent set by the Schloendorff case leads to a complicated relationship between ABA and the requirement that informed consent be received prior to the implementation of an intervention. If not preceded by appropriate informed consent, interventions that involve physical touch may qualify as battery (Marczyk & Wertheimer, 2001). Thus, the foreseeable use of restraints in a crisis, or even hand-over-hand prompting within routine teaching or assessment, may legally require informed consent.

Many behavior analytic interventions do not, however, require physical contact between the behavior analyst and the client. To the best of our knowledge it remains the case that, “[a]lthough informed consent is required for psychological treatments involving physical touching, the courts have not directly addressed the issue of whether informed consent must be obtained before other psychological services are rendered” (Marczyk & Wertheimer, 2001, p. 82). Indeed, there is effectively no case law that engages with the question of the requirements on informed consent in behavior analysis. For example, a Lexis-Nexis search for “‘informed consent’ AND ‘applied behavior analysis’” that includes cases in both federal and state courts returned only 11 hits,^4^ none of which substantively engage with the question of when (or if) informed consent is required for ABA interventions that do not involve physical contact. In some cases, it is unclear the extent to which informed consent is legally mandated for the provision of behavior analytic services.

This ambiguity is especially acute in the context of telehealth. In telehealth, it is common for a behavior analytic practitioner to coach a client caretaker who is then responsible for implementing therapy (Lerman et al., 2020). In this model, a caretaker—often a parent or guardian—is the one responsible for implementing therapy (Lerman et al., 2020). Given the grounding of informed consent case law in legal prohibitions against battery, the additional distance between the behavior analyst and the client introduced by telehealth adds further ambiguity regarding which aspects of behavior analytic practice are governed by the legal requirements on informed consent.

Despite this ambiguity, informed consent for the provision of behavior analytic services via telehealth is essential. In order for treatment plans to be implemented ethically and effectively, the caregiver must have a clear understanding of the end goal of the treatment as well as the potential outcomes and behavioral challenges that may arise throughout treatment (Pollard et al., 2017).

Despite the existence of substantial ambiguity in the case law regarding when informed consent must be provided as part of behavior analytic practice, in some cases statutory law offers definitive guidance. For example, the Individuals with Disabilities Education Act (IDEA) mandates the provision of informed consent prior to any assessment or intervention (Individuals with Disabilities Education Act, 2008). Informed consent is thus likely required for behavior analytic interventions that occur within the public-school context.

There remains, however, substantive ambiguity regarding those behavioral interventions for which informed consent is required. In the face of such ambiguity, behavior analysts may be wise to provide informed consent—adhering to the full breadth of legal requirements—for all interventions. There are at least three reasons that this is the case.

First, providing informed consent for all interventions guarantees that the legal requirements on informed consent are always met. If an ABA provider aims to only provide informed consent on those cases where it is legally necessary, they risk misidentification and may end up not providing informed consent when they are legally obligated to do so. By contrast, if a provider has a standing policy to provide informed consent regarding all interventions, they will never mistakenly fail to provide informed consent when it is legally required. Providing informed consent for all interventions is consequently a strategy that minimizes legal risk to ABA providers and violations of autonomy to clients.

Second, legal ambiguity regarding those interventions for which informed consent is required does not offer legal protection to behavior analysts. Prior to *Schloendorff* v. *Society of New York Hospital*, there was no established legal precedent that determined that medical interventions without consent were a form of battery. This lack of prior precedent did not prevent a finding against the Society of New York Hospital. Likewise, current ambiguity regarding when informed consent is legally mandated does not protect behavior analysts from a court finding that they should have, but did not, provide informed consent.

Finally, as discussed in the introduction, the provision of informed consent constitutes one of the core ethical bedrocks of contemporary biomedical ethics and is mandated by the *Ethics Code for Behavior Analysts*. Furthermore, informed consent is central to promoting clients’ self-determination and to treating “others with compassion, dignity, and respect” (BACB, 2020, p. 4); informed consent is key to involving “clients and relevant stakeholders throughout the service relationship” (p. 11); and informed consent is a necessary step in “support[ing] clients’ rights” (p. 13). Whether or not the provision of informed consent is legally required for all behavior analytic interventions, the universal provision of informed consent is ethically laudable and may be required by a number of principles and standards found in the *Ethics Code for Behavior Analysts*. Behavior analysts who strive to live up to the highest moral standard should thus consider making the provision of informed consent—corresponding with the most stringent of legal requirements—a staple of behavior analytic practice.

## Informed Consents’ Disclosure Requirements

Once the legal requirement for informed consent was established, a core question became: what information must be disclosed as part of the process of clinical informed consent? Many of the seminal legal cases regarding informed consent engaged with this question. As we will see, over the last century case law has trended toward ever more stringent disclosure requirements.

### The Community Disclose Standard: *DiFilippo* v. *Preston* (1961)

In April 1957, Anne DiFilippo sought out her doctor due to a visible lump that developed in her throat. Upon examination, her doctor determined that she had an enlarged thyroid gland, more commonly referred to as a goiter. She was advised to seek a second opinion and was referred to a surgeon for further examination. Dr. Daniel Preston concurred with the previous doctor's diagnosis of a goiter. Considering the pressure that was being put on her windpipe and the potential for the goiter to become malignant, Dr. Preston decided to forgo testing on the goiter and recommended surgery. Subsequent to the surgery Mrs. DiFilippo experienced paralysis of her vocal cords and had to get a tracheotomy, a procedure in which a hole is cut through the neck and into the windpipe and a tube is placed in the hole to allow breathing (*DiFilippo* v. *Preston*, 1961).

Mrs. DiFilippo argued that she was not given the opportunity to provide informed consent for the operation because (1) Dr. Preston did not inform her of the risk of vocal cord damage and because (2) “a surgeon owes to his patient a duty of disclosure of specific known risks. . .” (*DiFilippo* v. *Preston*, §549).

The court rejected this claim about the disclosure requirements on informed consent and, instead, held that informed consent only required that a health-care provider reveal as much information as is “the general practice with respect to such cases followed by the medical profession in the locality” (*DiFilippo* v. *Preston*, §549–550). Because it was not general practice in Mrs. Difilippo’s locality to inform patients about the potential risks to the vocal cords of a thyroidectomy, the court ruled that Dr. Preston had discharged his obligation to provide informed consent.

In making this ruling, the court relied on what has become known as the *Community Disclosure Standard* (sometimes also called the *Reasonable Professional Standard*). The Community Disclosure Standard holds that, during the process of informed consent, a health-care provider need only disclose as much information as other health-care providers in their community would disclose in similar circumstances.

### The Reasonable Person Standard: *Canterbury* v. *Spence* (1972)

In February 1959, Jerry Canterbury sought out treatment for ongoing back pain. Dr. William T. Spence suspected that Mr. Canterbury had a ruptured disk and recommended that Mr. Canterbury undergo a laminectomy, a surgery in which part or all of a vertebrae is removed. Mr. Canterbury “did not raise any objection to the proposed operation nor did he probe into its exact nature” (*Canterbury* v. *Spence*, 1972, §777). Furthermore, Mr. Canterbury “did not converse again with Dr. Spence prior to the operation” (*Canterbury* v. *Spence*, 1972, §777). Two days following the surgery Mr. Canterbury suffered a fall in the hospital due to a miscommunication on postoperative instructions. The combination of the high-risk surgery and the fall rendered Mr. Canterbury paralyzed from the waist down. Despite an additional emergency surgery to correct his paralysis, Mr. Canterbury remained unable to walk unassisted and experienced urinary incontinence and paralysis of his bowels. Mr. Canterbury brought Dr. Spence to court, arguing that Dr. Spence had failed to provide informed consent because the risk of paralysis and other complications were not disclosed to Mr. Canterbury. Dr. Spence stated that he did not disclose the risks to the patient because he did not want the potential risks to deter him from undergoing the surgery (*Canterbury* v. *Spence*, 1972).

In its ruling, the court established what is now known as the *Reasonable Person Standard*. This standard requires that, as part of the process of informed consent, a health-care provider disclose all of the information that a “reasonable” person would want to know. The notion of a “reasonable person” remains somewhat slippery but is perhaps most straightforwardly understood as an empirical construct, i.e., the “reasonable person” is roughly the average person (Miller & Perry, 2012). Despite the somewhat ambiguous nature of the construct, as the court noted in *Canterbury* v. *Spence*, the use of the “reasonable person” as a measuring stick of permissible behavior is common in negligence jurisprudence (*Canterbury* v. *Spence*).

By indexing disclosure requirements to the reasonable person, the Reasonable Person Standard offers general guidance regarding what information behavior analysts should disclose during the process of clinical informed consent. In the words of a 1903 English Court, the reasonable person is “the man [sic] on the Clapham omnibus” (*Bolam* v. *Friern Hosp. Management Comm.*). Or, in the parlance of our times, the “person riding the subway” or “the person commuting to work.” The Reasonable Person Standard thus sets an egalitarian norm for informed consent disclosure. No special expertise is required to know whether some particular information should be disclosed during the process of information consent; we are all (roughly) the “reasonable person.” Thus, in determining what information should be disclosed during the process of clinical informed consent, the behavior analyst need only ask themselves: “were I in the client’s position, what would I want to know before making this decision?”

In *Canterbury* v. *Spence*, the Court went beyond merely establishing the Reasonable Person Standard as criterial for information disclosure during informed consent. The Court further specified information that the reasonable person would want to know before making a choice regarding some intervention, establishing three requirements that are notably missing from the disclosure requirements enumerated in the *Ethics Codes for Behavior Analysts*. First, *Canterbury* v. *Spence* requires that informed consent include a discussion of alternative interventions. For example, informed consent for planned ignoring is incomplete without a discussion of differential reinforcement of alternative behavior and other available treatment options.

Second, *Canterbury* v. *Spence* requires that informed consent include a discussion of potential outcomes if no treatment is offered. As per the Reasonable Person Standard, the process of informed consent for behavior analytic interventions is not complete until a client and behavior analyst have discussed the potential outcomes of the choice to forgo an intervention entirely.

Finally, *Canterbury* v. *Spence* requires that informed consent be provided for *individual* interventions: health-care providers must disclose all material information regarding a “medical technique” (*Canterbury* v. *Spence*, 1972, §788). In line with this aspect of *Canterbury* v. *Spence*, behavior analysts should get informed consent for every piece of a treatment package; it is not adequate to get informed consent for, for example, 30 hr per week of EIBI. Rather, behavior analysts must get informed consent for individual components of the treatment package. That is, behavior analysts must get informed consent for implementing escape extinction within the context of tabletop academic work; behavior analysts must get informed consent for the differential reinforcement of alternative behavior within the context of treating stereotypy; behavior analysts must get informed consent for the use of contingent reinforcement for accurate responding within the context of discrete trial training, etc.

Therapeutic contexts are often fluid, requiring that behavior analysts respond flexibly to a client’s changing motivations and behavioral repertoire. It may often not be feasible for behavior analysts to engage in the informed consent process at every choice point for *in vivo* therapy. This does not, however, invalidate the need for informed consent. Rather, the informed consent process should include a discussion of the range of foreseeable paths the intervention might take. For example, a behavior analyst might discuss the potential for the emergence of novel problem behavior within the context of an extinction procedure, the various intervention options given this potential outcome, the pros and cons of each intervention option, and the indicators that the behavior analyst takes to be relevant to making the relevant clinical decisions.

The Reasonable Person Standard should guide this discussion. Foreseeable behavioral outcomes that are both unlikely and low risk need not be discussed; the reasonable person would not take such outcomes to be relevant to making intervention decisions. By contrast, unlikely but potentially harmful behavioral outcomes should be discussed, along with the various intervention options that a behavior analyst might implement in response, and the pros and cons and such options. In the language of the Court:

A very small chance of death or serious disablement may well be significant; a potential disability which dramatically outweighs the potential benefit of the therapy or the detriments of the existing malady may summons discussion with the patient. (*Canterbury* v. *Spence,* 1972, *§*788)

Though the shift to the Reasonable Person Standard demanded by *Canterbury* v. *Spence* constitutes one of the most fundamental shifts in the legal requirements on informed consent, there is no universal answer regarding which standard—the Reasonable Person Standard or the Community Disclosure Standard—a health-care provider is required to follow. Rather, the legal requirements vary state-to-state. Studdert et al. (2007) found that about half the states rely on the Reasonable Person Standard, about half the states rely on the Community Disclosure Standard, and that two states use a hybrid system. The result is that the legal requirements of informed consent in clinical behavior analysis can vary significantly, depending on the state in which a behavior analyst practices.

It is likely that, in those states that continue to rely on the Community Disclosure Standards, the disclosure requirements enumerated in the *Ethics Code for Behavior Analysts* (Behavior Analyst Certification Board, 2020) would serve as guideposts for the disclosure practices common amongst behavior analysts (Dolgin, 2010). As such, in approximately half of the states, the requirements on informed consent included in the *Ethics Code for Behavior Analysts* likely determine the information that behavior analysts are legally required to disclose during the process of informed consent. As we will continue to see, in those states that rely on the Reasonable Person Standard, the legal requirements on informed consent are likely substantively more stringent than those outlined in the *Ethics Code for Behavior Analysts*.

This variation in legal standards presents a challenge to the geographic uniformity of the quality of behavior analysis. Clients in states that rely on the Reasonable Person Standard may be legally owed a greater depth of disclosure than clients in states that rely on the Community Disclosure Standard. Other health-care disciplines have navigated this challenge by including the Reasonable Person Standard within their practice guidelines (Dolgin, 2010). This effectively collapses the two standards, as it establishes that community practice is to disclose all of the information that a reasonable person would want to know (Dolgin, 2010).

### *Gates* v. *Jenson* (1979): Expanding Disclosure Requirements to Include Alternative Assessments and Future Risks

Much of case law related to informed consent builds on *Canterbury* v. *Spence* by further specifying the information that a reasonable person would want to know in making a health-care decision. In this manner, the disclosure requirements on informed consent have gradually expanded. Because this expansion of disclosure requirements builds on the Reasonable Person Standard, these expanded disclosure requirements are likely not operative for those behavior analysts that practice in a state that still utilizes the Community Disclosure Standard.

*Gates* v. *Jenson* (1979) is an important case in the gradual expansion of the informed consent disclosure requirements. In April 1972, Mrs. Elisabeth Gates sought out her ophthalmologist, Dr. James Hargiss, because she was experiencing blurred vision and difficulty focusing her sight. She was an older woman who had a preexisting condition that put her at high risk for glaucoma. Dr. Hargiss tested the pressure in her eyes and noted that it was quite high. Dr. Hargiss then checked her optic nerves without dilating her eyes. His exam was unremarkable and he ran no other tests for glaucoma. Dr. Hargiss told Mrs. Gates that everything was alright and that her troubles likely stemmed from her inability to adjust to her new contact lenses. Despite going back to various eye doctors over the next 2 years, Mrs. Gates never had her eyes checked under dilation due to Dr. Hargriss’s diagnosis of eye sensitivity. When her eyes were finally checked under dilation it was discovered that she did in fact have glaucoma and her 20/20 vision had deteriorated to 20/200, making her functionally blind (*Gates* v. *Jenson*, 1979).

Mrs. Gates sued Dr. Hargriss, alleging, among other things, failure to provide adequate informed consent. In *Gates* v. *Jenson*, the court ruled for Mrs. Gates and expanded the precedent established under *Canterbury* v. *Spence*, adding three additional disclosure requirements: (1) the requirement that, when discovered, clients be informed of abnormalities in their bodies; (2) the requirement that, when applicable, clients be informed of having a high risk for future disease; and (3) the requirement that clients be informed of alternative diagnostic procedures. Of these three requirements, the first is not immediately relevant to behavior analysts, as behavior analysts do not diagnose bodily abnormalities. The latter two are, however, likely relevant to the clinical practice of behavior analysis.

The requirement that clients be informed of alternative diagnostic procedures may have the greatest implications for the practice of informed consent in clinical behavior analysis. For example, *Gates* v. *Jenson* has implications for the practice of informed consent regarding functional behavioral assessments (FBAs). A wide range of FBAs are available, varying from indirect assessments (Floyd et al., 2005) to the (traditional) functional analysis (Iwata et al., 1982/1994). Each FBA comes with its own costs and benefits. A behavior analyst that received consent for an indirect assessment but failed to discuss the option of a functional analysis could be liable for any harms resulting from a failure to correctly identify the function of a behavior. Likewise, a behavior analyst that received consent for a (traditional) functional analysis but failed to discuss the costs and benefits of indirect assessments, nonexperimental observational assessments, and the brief functional analysis (Northup et al., 1991), could find themselves liable for any harm resulting from a client undergoing the (traditional) functional analysis.

*Gates* v. *Jenson* should not, however, be read as requiring a blanket requirement that behavior analysts *always* discuss alternative assessment options. The *materiality* condition is key to the Reasonable Person Standard. Information is material when a reasonable person “would be likely to attach significance to the risk or cluster of risks in deciding whether or not to forgo the proposed therapy [or assessment]” (*Canterbury* v. *Spence*, 1972, §787).

It is unclear that there is any material difference between, for example, a forced-choice preference assessment and a multiple-stimulus without replacement preference assessment. Furthermore, although there is likely a material difference between the traditional functional analysis and the brief functional analysis, there may not be a material difference between different ways of implementing an indirect assessment. Thus, though *Gates* v. *Jenson* expanded disclosure requirements, the materiality condition introduced by the Reasonable Person Standard puts meaningful constraints on this expansion.

*Gates* v. *Jenson* further demands that clients be informed of their risk of future disease. This disclosure requirement also has implications for the practice of informed consent in clinical behavior analysis. For example, the informed consent process for autistic children with restricted eating habits should include a discussion of the potential long-term harms associated with excessive food selectivity (Peterson et al., 2019; Peterson et al., 2016). Likewise, the informed consent process for children with autism who resist teeth brushing should include a discussion of the long-term harms associated with poor oral hygiene (Carter et al., 2019; Cullinan et al., 2009; Iwata & Becksfort, 1981). In those cases where a behavior analyst fails to discuss a client’s high-risk for future disease during the informed consent process, the behavior analyst may fail to fulfill their disclosure requirements and thereby put themselves at legal jeopardy.

Behavior analysts may not feel prepared to discuss the long-term sequelae of food selectivity or poor oral hygiene. In such cases, behavior analysts may wish to consult the academic literature or a comparatively expert professional, e.g., a dentist. It should also be noted that the materiality condition on informed consent remains relevant in this context. For example, raising awareness that food selectivity may not go away without intervention (Peterson et al., 2016), and that food selectivity can lead to serious medical conditions with the potential to substantially diminish quality of life and life expectancy (Peterson et al., 2019; Peterson et al., 2016) may be adequate to fulfill the relevant disclosure requirements on informed consent. Thus, although a more detailed discussion of the health impacts of obesity and Type 2 diabetes likely falls outside of the scope of practice for many behavior analysts, it is unclear if such details are material to the decision to implement interventions for food selectivity.

### *Truman* v. *Thomas* (1980): The Risks of Foregoing Assessment and the Relevance of Unique Concerns

Dr. Claude Thomas served as Mrs. Rena Truman’s primary care physician for 6 years. During this time Dr. Thomas recommended to Mrs. Truman that she receive regular pap smears but did not disclose the risks associated with foregoing a pap smear. Mrs. Truman declined to have a pap smear. Several years later Mrs. Truman was diagnosed with cervical cancer from which she ultimately died. Mrs. Truman’s children sued Dr. Thomas on the grounds that, because Dr. Thomas did not disclose the risks associated with not undergoing regular pap smears, he failed to provide adequate informed consent. The Court ruled against Dr. Thomas and expanded the disclosure requirements under the Reasonable Person Standard by requiring that health-care providers disclose the risks of choosing *not* to undergo an assessment (*Truman* v. *Thomas*, 1980).

In their ruling, the Court further wrote: “If the physician knows or should know of a patient's unique concerns or lack of familiarity with medical procedures, this may expand the scope of required disclosure” (*Truman* v. *Thomas*, 1980, § 291). Though not the emphasis of the Court’s ruling, this component of the Court’s decision suggests an important expansion of the information that must be disclosed as part of the informed consent process. It is not merely enough to provide all of the information that the Reasonable Person would want to know. Rather, disclosure must further be governed by what the health-care provider knows, or should know, about a “patient’s unique concerns.”

This additional requirement has important implications for the clinical process of informed consent in behavior analysis. Consider, for example, a client who has undergone intervention to eliminate self-injurious behavior. For such a client, the informed consent process for an extinction procedure is likely not complete unless a client has been advised of the risk for the resurgence of behavior that had previously been treated (Doughty & Oken, 2008); a similar disclosure would not be required for a client that has not previously undergone intervention to eliminate a target behavior.

Likewise, clients may have “unique concerns” that may require additional disclosures on the part of behavior analysts. For example, the regular use of a reinforcer in a clinical context may lead a client to satiate on a reinforcer and thus potentially reduce a client’s motivating operation for that same reinforcer when they return to the home context at the end of the day. If access to the reinforcer plays an important role in how parents mediate problem behavior at home, it is important that behavior analysts discuss the potential impacts on satiation when using the reinforcer in therapy. When making their health-care decisions, the reasonable person is unlikely to care about the potential of becoming “bored” of using an iPad. Nonetheless, due to the unique concerns relevant to some clients, this may be something that behavior analysts are required to disclose as part of the process of informed consent.

### *Johnson* v. *Kokemoor* (1996): Disclosing Behavior Analysts’ Level of Expertise

In 1994 Donna Johnson went to Dr. Kokemoor regarding the removal of an aneurysm. He told her that the risk of serious impairment was around 2% for the surgery. When she inquired about his experience level, Dr. Kokemoor told Mrs. Johnson that he had done the procedure dozens of times. However, Dr. Kokemoor had only removed 12 such aneurysms and only 2 of those had been in the same high-risk position as Mrs. Johnson’s aneurysm. Mrs. Johnson suffered complications following the procedure including incomplete quadriplegia and blindness. Mrs. Johnson sued, alleging that Dr. Kokemoor had failed to provide adequate informed consent (*Johnson* v. *Kokemoor*, 1996).

The Court ruled in Mrs. Johnson’s favor, introducing a major expansion of the disclosure requirements under the Reasonable Person Standard. In addition to the preestablished disclosure requirements, *Johnson* v. *Kokemoor* requires that health-care providers disclose, when it would be relevant to how a Reasonable Person would make their decision, (1) their lack of experience with a procedure; (2) their lack of success with a procedure, as compared to their peers; and (3) the existence of alternative health-care providers able to (better) perform the same intervention.

Particularly given the rate at which new behavior analysts are entering the field (Rosenberg & Schwartz, 2019), the ruling in *Johnson* v. *Kokemoor* has potentially profound implications for the clinical process of behavior analytic informed consent. For example, a behavior analyst with limited experience performing functional analyses may need to disclose this fact as part of the clinical informed consent process.

Whereas the ruling in *Johnson* v. *Kokemoor* may seem worrisome to behavior analysts who are comparatively new in the field, it does little to reshape our understanding of best practice. Behavior analysts only practice within their scope of competence, do not accept clients whose needs outstrip their scope of competence, and seek out appropriate supervision before moving forward with an assessment or intervention with which they have limited familiarity (BACB, 2020). Though the *Ethics Code for Behavior Analysts* does not require that behavior analysts disclose limited experience with an intervention as part of the informed consent process, the *Code* nonetheless introduces a number of guardrails that should prevent circumstances from arising in which such a disclosure would be required.

## Discussion

The case law governing informed consent in America is both complicated and voluminous. Our goal in this section is to provide a general guide for clinical informed consent which behavior analysts can use to develop their own informed consent practices. Legal standards for informed consent vary depending on where a behavior analyst practices. Insofar as it is possible to give a general guide to informed consent in clinical behavior analysis, the guide must conform to the most stringent legal requirements as only this approach can minimize legal jeopardy for all behavior analysts.

It should be stressed that the following guide aims to capture some of the minimum legal requirements for clinical informed consent. The following guide should not, however, be taken to enumerate the conditions *sufficient* to meet the legal requirements on clinical informed consent. It thus remains possible that a behavior analyst may fail to fulfill the legal requirements on clinical informed consent even though they rely on the following guidance. (Behavior analysts may also find Appendix 1 useful as a tool when preparing for informed consent conversations.)

*When is informed consent required?*

- Informed consent should be provided whenever there are facts about an intervention or assessment that are relevant to how a reasonable person would make their decision.
- Informed consent must be provided for individual interventions and individual assessments. Informed consent cannot be provided for a package of interventions, for example, 30 hr a week of EIBI.

*What should be disclosed during the process of informed consent?*

The Reasonable Person Standard should govern a behavior analysts’ disclosures during the process of informed consent. The history of case law regarding informed consent and the Reasonable Person Standard involves an ever-expanding list of disclosure requirements. Whereas case law establishes minimum disclosure requirements during the informed consent process, as per the Reasonable Person Standard, behavior analysts should additionally disclose any information that they think might change a client’s decision regarding consent for an assessment or intervention.

The courts have ruled that the Reasonable Person Standard requires the disclosure of all of the following:

• The risks and benefits of an assessment or treatment;

- The risks and benefits of alternative assessments or treatments;
- The risks and benefits of foregoing assessment or treatment altogether;
- A patient’s risk for future disease;
- Where applicable, a behavior analyst’s limited experience with an assessment or intervention;
- Where applicable, a behavior analyst’s low rate of success with an assessment or intervention;
- Where applicable, the availability of other providers who are comparatively more experienced with the assessment or intervention.

Disclosure of the above should be guided by the materiality condition. In most cases, risks and benefits that are too minimal to influence the decision-making of a reasonable person need not be disclosed. In those instances where a client has “unique concerns,” disclosures should be guided by what the client would take to be important for their decision, rather than by what the reasonable person would take to be important (*Truman* v. *Thomas*, 1980). Finally, risks that are not inherent to an assessment or intervention but that could result from negligence or malpractice do not need to be disclosed (*Gilmartin* v. *Weinreb*, 1999).

## Future Directions

There are important differences in the legal standards that govern informed consent and the discussion of informed consent found in leading behavior analytic ethics resources. It is, however, unclear how this difference plays out in actual behavior analytic practice. Are behavior analysts following the informed consent guidance found in leading behavior analytic ethics resources, are they following the relevant legal standards, or are they perhaps following both? Empirical work is needed to document the actual practice of informed consent in therapeutic and behavior analytic contexts and to assess the need for future work designed to further familiarize behavior analysts with the relevant legal standards.

The guidance regarding informed consent offered herein remains schematic and thus of potentially limited value. Behavior analysts may, for example, still wonder: “what do I need to disclose in the informed consent process for a functional analysis?” or “what do I need to disclose in the informed consent process for implementing picture exchange?” Knowing that one must generally disclose the risks and benefits of an intervention and the risks and benefits of alternative interventions offers little guidance regarding what concrete information a behavior analyst ought to share during the informed consent process for a functional analysis or for the use of picture exchange.

Important work remains to be done in this area. For example, knowledge regarding adverse effects of pediatric autism interventions remains limited (Bottema-Beutel et al., 2021). In general, it is unclear if there is widespread agreement among behavior analysts regarding the risks and benefits of a range of interventions. There is thus a need for (1) projects that aim to further our understanding of the benefits and risks of various intervention and (2) subsequent projects that produce concrete guidance, based on our understanding of the risks and benefits of various interventions, regarding what behavior analysts should disclose before implementing specific types of interventions.

Finally, legal and ethical standards often come apart (Beauchamp & Childress, 2019). The fact that something is illegal does not entail that it is unethical. Likewise, the fact that something is unethical does not entail that it is illegal. Future work investigating the overlap of the legal and ethical standards regarding informed consent in therapeutic and behavior analytic contexts can advance our understanding of the ethical obligations of the behavior analyst with regard to informed consent. In particular, because contemporary case law regarding informed consent is centered on physicians, consideration of the differences in the ethical obligations between behavior analysts and physicians as regards informed consent may prove to be a fertile area for future investigation.

## Conclusion

Case law regarding informed consent in the United States puts a number of requirements on informed consent that go beyond the enumerated requirements on informed consent found in the *Ethics Code for Behavior Analysts* and in other ethics texts written for behavior analytic audiences. Behavior analysts that fail to live up to the legal standards established in case law may put themselves in legal jeopardy.

Because our federalist system has led to a patchwork of legal requirements governing informed consent, not all of the precedents discussed above will be legally binding for every behavior analyst. This complication is further compounded by the fact that the courts have yet to determine if informed consent is required for interventions that do not involve physical touch. Nonetheless, in order to uphold the highest ethical standards, in order to simplify organizational procedures regarding informed consent, and because existing case law in other jurisdictions can serve as an indication of how courts will rule on similar cases in one’s own jurisdiction, it is advisable that behavior analysts uphold the most stringent set of minimum legal requirements on informed consent that has been established by the courts.

### Supplementary Information

ESM 1 (DOCX 11 kb)

## Data Availability

Data sharing not applicable to this article as no datasets were generated or analyzed during the current study.

## Footnotes

1 “Parentalism” is a gender-neutral form of “paternalism” (Suber, 1999).

2 “Decisional capacity” is a clinical term and is determined by health-care providers whereas “decisional competence” is a legal term and is determined by courts (Graber, 2021). Even seminal articles on capacity/competence treat the two as being interchangeable (Graber, 2021; see, e.g., Appelbaum, 2007). There are, however, good reasons to think that the distinction between capacity and competence has profound implications in therapeutic contexts, especially as it relates to clients with intellectual disability (Graber, 2023). The distinction between competence and capacity and the implications for that distinction for behavior analytic practice, especially as it involves assent, are nuanced issues that are in need of greater attention in the behavior analytic literature. That is not, however, a project that we can undertake here.

3 The relevant case law is not discussed in this article but is currently only operative in Pennsylvania (Lynch et al., 2018).

4 Search performed on May 3, 2023.

## References

[CR1] Appelbaum PS (2007). Assessment of patients' competence to consent to treatment. New England Journal of Medicine.

[CR2] Bailey, J., & Burch, M. (2022). *Ethics for behavior analysts* (4^th^ ed.). Routledge.

[CR3] Beauchamp, T., & Childress, J. (2019). *Principles of biomedical ethics* (8th ed.). Oxford University Press.

[CR4] Behavior Analyst Certification Board. (2020). *Ethics code for behavior analysts*. https://bacb.com/wp-content/ethics-code-for-behavior-analysts/

[CR5] Beirne, A., & Sadavoy, J. A. (2022). *Understanding ethics in applied behavior analysis: Practical applications* (2nd ed.). Routledge. *Bolam* v. *Friern Hosp. Management Comm*., [1957] 1 W.L.R. 582, 586-88.

[CR6] Bottema-Beutel K, Crowley S, Sandbank M, Woynaroski TG (2021). Adverse event reporting in intervention research for young autistic children. Autism.

[CR7] Breaux CA, Smith K (2023). Assent in applied behaviour analysis and positive behaviour support: ethical considerations and practical recommendations. International Journal of Developmental Disabilities.

[CR8] Brodhead, M., Cox, D., & Quigley, S. (2018). *Practice ethics for effective treatment of autism spectrum disorder* (2nd ed.). Academic Press.

[CR9] Brodhead, M., Cox, D., & Quigley, S. (2022). *Practice ethics for effective treatment of autism spectrum disorder* (3rd ed.). Academic Press.

[CR10] Brody H (1989). Transparency: Informed consent in primary care. Hastings Center Report.

[CR11] Canterbury v. Spence, 464 F.2d 772 (D.C. Cir. 1972).

[CR12] Carter L, Harper JM, Luiselli JK (2019). Dental desensitization for students with autism spectrum disorder through graduated exposure, reinforcement, and reinforcement-fading. Journal of Developmental & Physical Disabilities.

[CR13] Cassell EJ (2000). The principles of the Belmont report revisited: How have respect for persons, beneficence, and justice been applied to clinical medicine?. Hastings Center Report.

[CR14] Cullinan MP, Ford PJ, Seymour GJ (2009). Periodontal disease and systemic health: current status. Australian Dental Journal.

[CR15] DiFilippo v. Preston, 173 A.2d 333, 53 Del. 539 (1961).

[CR16] Dolgin JL (2010). The legal development of the informed consent doctrine: Past and present. Cambridge Quarterly of Healthcare Ethics.

[CR17] Doughty AH, Oken G (2008). Extinction-induced response resurgence: A selective review. The Behavior Analyst Today.

[CR18] Emanuel EJ, Emanuel LL (1992). Four models of the physician-patient relationship. Journal of the American Medical Association.

[CR19] Eyal, N. (2019). Informed consent. *The Stanford encyclopedia of philosophy*. https://plato.stanford.edu/archives/spr2019/entries/informed-consent/

[CR20] Flowers, J., & Dawes, J. (2023). Dignity and respect: Why therapeutic assent matters. *Behavior analysis in practice* (pp. 1–8). 10.1007/s40617-023-00772-6 PMC10700258 38076752

[CR21] Floyd RG, Phaneuf RL, Wilczynski SM (2005). Measurement properties of indirect assessment methods for functional behavioral assessment: A review of research. School Psychology Review.

[CR22] Gates v. Jensen, 595 P.2d 919, 92 Wash. 2d 246 (1979).

[CR23] *Gilmartin* v. *Weinreb*, 324 N.J. Super. 367, 371, 735 A.2d 620, 622, 1999 N.J. Super. LEXIS 299, *1 (App.Div. August 27, 1999).

[CR24] Graber A (2021). Justifying risk-related standards of capacity via autonomy alone. Journal of Medical Ethics.

[CR25] Graber, A. (2023). *Free Britney! Capacity, competence, and consent for those with diminished decision-making ability.* Bioethics Grand Rounds.

[CR26] Grimm DA (2007). Informed consent for all!. No exceptions. New Mexico Law Review.

[CR27] Individuals with Disabilities Education Act. 20 U.S.C. 300 § 300 (2008). https://sites.ed.gov/idea/regs/b/d/300.300

[CR28] Iwata BA, Becksfort CM (1981). Behavioral research in preventive dentistry: Educational and contingency management approaches to the problem of patient compliance. Journal of Applied Behavior Analysis.

[CR29] Iwata BA, Dorsey MF, Slifer KJ, Bauman KE, Richman GS (1994). Toward a functional analysis of self-injury. Journal of Applied Behavior Analysis.

[CR30] Johnson v. Kokemoor, 545 N.W.2d 495, 199 Wis. 2d 615, 199 Wis. 3d 615 (1996).

[CR31] Katz, J. (1984). *The silent world of doctor and patient*. Free Press.

[CR32] Lerman, D. C., O’Brien, M. J., Neely, L., Call, N. A., Tsami, L., Schieltz, K. M., ... & Cooper-Brown, L. J. (2020). Remote coaching of caregivers via telehealth: Challenges and potential solutions. *Journal of Behavioral Education, 29*, 195–221. 10.1007/s10864-020-09378-2 PMC9455948 36093285

[CR33] Lynch HF, Joffe S, Feldman EA (2018). Informed consent and the role of the treating physician. New England Journal of Medicine.

[CR34] Marczyk GR, Wertheimer E (2001). The bitter pill of empiricism: Health maintenance organizations, informed consent and the reasonable psychotherapist standard of care. Villanova Law Review.

[CR35] Miller AD, Perry R (2012). The reasonable person. New York University Law Review.

[CR36] Northup J, Wacker D, Sasso G, Steege M, Cigrand K, Cook J, DeRaad A (1991). A brief functional analysis of aggressive and alternative behavior in an out clinic setting. Journal of Applied Behavior Analysis.

[CR37] Peterson KM, Piazza CC, Ibañez VF, Fisher WW (2019). Randomized controlled trial of an applied behavior analytic intervention for food selectivity in children with autism spectrum disorder. Journal of Applied Behavior Analysis.

[CR38] Peterson KM, Piazza CC, Volkert VM (2016). A comparison of a modified sequential oral sensory approach to an applied behavior-analytic approach in the treatment of food selectivity in children with autism spectrum disorder. Journal of Applied Behavior Analysis.

[CR39] Pollard JS, Karimi KA, Ficcaglia MB (2017). Ethical considerations in the design and implementation of a telehealth service delivery model. Behavior Analysis: Research & Practice.

[CR40] Rosenberg NE, Schwartz IS (2019). Guidance or compliance: What makes an ethical behavior analyst?. Behavior Analysis in Practice.

[CR41] *Salgo* v. *Leland Stanford Jr. University Board of Trustees*, 154 Cal. App. 2d 560, 578, 317 P.2d 170, 181, 1957 Cal. App. LEXIS 1667, *34-35 (Cal. App. 1st Dist. October 22, 1957).

[CR42] *Schloendorff* v. *Soc'y of N.Y. Hospital*, 211 N.Y. 125, 129-130, 105 N.E. 92, 93, 1914 N.Y. LEXIS 1028, *8 (N.Y. April 14, 1914).

[CR43] Shuster E (1997). Fifty years later: the significance of the Nuremberg Code. New England Journal of Medicine.

[CR44] Studdert DM, Mello MM, Levy MK, Gruen RL, Dunn EJ, Orav EJ, Brennan TA (2007). Geographic variation in informed consent law: two standards for disclosure of treatment risks. Journal of Empirical Legal Studies.

[CR45] Suber P, Gray CB (1999). Parentalism. *Philosophy of law: An encyclopedia*.

[CR46] Susha D, Najdowski AC (2021). *A workbook of ethical case scenarios in applied behavior analysis*.

[CR47] *Truman* v. *Thomas*, 27 Cal. 3d 285, 292, 611 P.2d 902, 906, 165 Cal. Rptr. 308, 312, 1980 Cal. LEXIS 175, *10 (Cal. June 9, 1980).

[CR48] Wasserman JA, Navin MC, Vercler CJ (2019). Pediatric assent and treating children over objection. Pediatrics.

