# Supplementary material for: Clinical Informed Consent and ABA
Source: Behav Anal Pract. 2024 Jan 8;17(2):389–400. doi: 10.1007/s40617-023-00902-0 (PMC11219615; doi:10.1007/s40617-023-00902-0)
Supplement: Supplementary file 1 — (DOCX 11 kb) [file 40617_2023_902_MOESM1_ESM.docx]

**Appendix 1: Clinical Informed Consent Inventory**

- Informed consent is a core clinical skill that cannot be adequately captured in a checklist or document. The following informed consent inventory may be useful in helping the behavior analyst prepare for the informed consent conversation; however, the relevant information should be disclosed over the course of a naturalistic conversation with the client. As such, *we do not recommend using the following inventory as a centerpiece of the informed consent conversation.*
- The following inventory aims to capture the requirements on informed consent established by the Reasonable Person Standard; it should be used in addition to, *not* as a replacement for, informed consent documents based on The Ethics Code for Behavior Analysts.
- The following informed consent inventory does not constitute legal advice or guidance; behavior analysts in need of legal guidance regarding clinical informed consent should seek the services of a licensed attorney.

| **Informed Consent Inventory** | | |
| --- | --- | --- |
| Initial Questions | | |
|  | **Yes** | **No** |
| **Are you seeking informed consent regarding a treatment package?** | Informed consent must be provided regarding individual interventions and assessments. | Good! Informed consent should be obtained for each proposed intervention and/or assessment. |
| **Are there facts about the intervention or assessment that are relevant to how a reasonable person would make their decision?** | Informed consent is required before moving forward with the proposed intervention or assessment. | If there are no facts about the intervention or assessment that a reasonable person would want to know in order to make their decision, informed consent is not required before moving forward.  Given the potential legal and ethical risks, a high degree of confidence is recommended before choosing to forego informed consent. |

| Disclosure Inventory: Prior to assessment/intervention  What should be disclosed as part of the informed consent process? | | |
| --- | --- | --- |
| **Proposed Intervention/Assessment** | Potential benefits:**^†^** | Potential risks:**^†^** |
|  | Extent of experience with intervention/assessment: | |
|  | Rate of success with intervention/assessment: | |
|  | Availability of alternative provider with greater experience or better rates of success: | |
| **Alternative Intervention/Assessment #1^‡^** | Potential benefits: | Potential risks: |
|  | Extent of experience with intervention/assessment: | |
|  | Rate of success with intervention/assessment: | |
|  | Availability of alternative provider with greater experience or better rates of success: | |
| **Alternative Intervention/Assessment #2** | Potential benefits: | Potential risks: |
|  | Extent of experience with intervention/assessment: | |
|  | Rate of success with intervention/assessment: | |
|  | Availability of alternative provider with greater experience or better rates of success: | |
| **Alternative Intervention/Assessment #3** | Potential benefits: | Potential risks: |
|  | Extent of experience with intervention/assessment: | |
|  | Rate of success with intervention/assessment: | |
|  | Available alternative providers with greater experience or better rates of success: | |
| **Forgoing Intervention/Assessment** | Potential benefits: | Potential risks: |

**^†^** ^Behavior analysts need only disclose those risks, benefits, and risks for future disease that either (i) a reasonable person would take into account while making their decision or (ii) information that the client, due to their unique circumstances, would take to be important when making their decision.^

**^‡^** ^There is no in principle limit to the number of alternative interventions or assessments that should be included in the informed consent conversation. In rare cases, the proposed intervention may be the only one that a reasonable person would consider, in which case no other alternative interventions or assessments would need to be discussed. In other instances, there may be well more than three alternatives that a reasonable person would consider, all of which would need to be included in the informed consent conversation. The number of alternative interventions/assessments included in this document should not be taken as a guide to the number of interventions/assessments that should be included in the informed consent conversation.^

| Disclosure Inventory: After assessment/intervention  What should be disclosed as part of the informed consent process? | |
| --- | --- |
| **Risk of Future Disease and/or Problem Behavior** | During the intervention/assessment, were there any indications of risk of future disease for the client:**^†^** |
